# Immunoglobulin A Nephropathy with Erythrocytosis: An Unusual Association

**DOI:** 10.7759/cureus.13437

**Published:** 2021-02-19

**Authors:** Piratheepan Navaradnam, Vathulan Sujanitha, Navaneethakrishnan Suganthan, Thaneswary Sooriyakumar, Malaravan Muthusamy

**Affiliations:** 1 Medicine, Teaching Hospital Jaffna, Jaffna, LKA; 2 Medicine, Postgraduate Institute of Medicine, University of Colombo, Colombo, LKA; 3 Medicine, University of Jaffna, Jaffna, LKA; 4 Hematology, Teaching Hospital Jaffna, Jaffna, LKA; 5 Ophthalmology, Teaching Hospital Jaffna, Jaffna, LKA

**Keywords:** iga nephropathy, polycythemia, proteinuria, erythrocytosis

## Abstract

Immunoglobulin A (IgA) nephropathy is the commonest type of glomerular nephritis worldwide and is one of the leading causes of chronic kidney disease. Erythrocytosis is known to be associated with IgA nephropathy and other renal manifestations. We report a case of a 35-year-old male with grade 3 hypertensive retinopathy, erythrocytosis, and heavy proteinuria with an impaired renal function who was diagnosed as a case of erythrocytosis with IgA nephropathy and secondary focal segmental glomerulosclerosis. An extensive evaluation and prompt interventions significantly improved the outcome in this patient.

## Introduction

Erythrocytosis is known to be associated with immunoglobulin A (IgA) nephropathy, focal segmental glomerular sclerosis (FSGS), mesangial proliferative glomerular nephritis (MPGN), and rapidly progressive glomerular nephritis (RPGN) [1]. The mechanism of renal involvement is complex and involves the formation of vascular microthrombi and glomerular capillary occlusion, and thereby reduction in glomerular filtration rate (GFR) and tissue ischemia [1]. Here we report a case of erythrocytosis associated with IgA nephropathy and secondary FSGS leading to hypertension and early chronic kidney disease in the absence of positive myeloproliferative neoplasm panel (MNP) and very low plasma erythropoietin level.

## Case presentation

**Figure 1:**
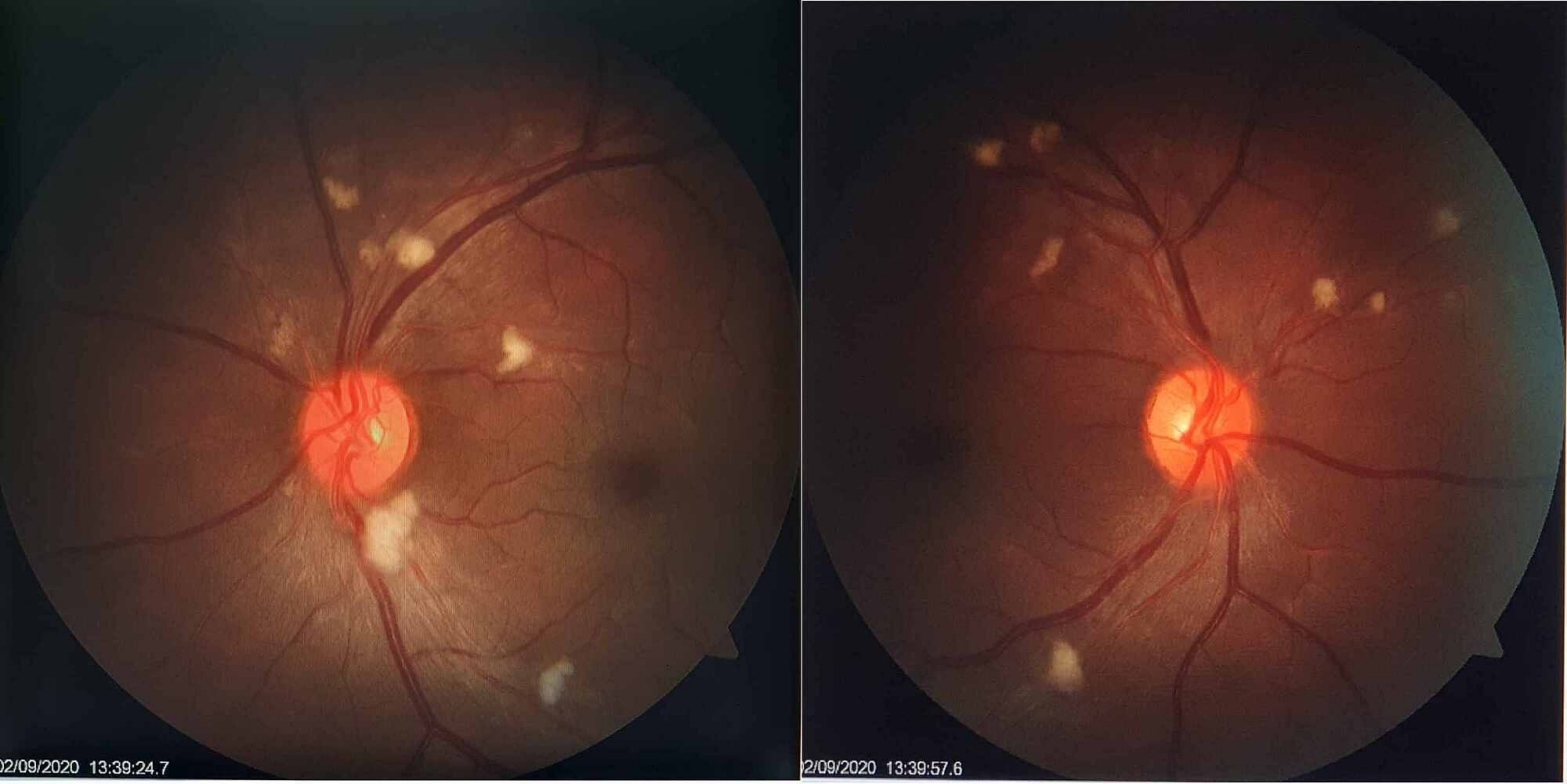
Ophthalmoscopic examination shows congested retinal vessels with grade 2 hypertensive retinopathy.

A 35-year-old previously healthy nonsmoker presented to the Emergency Department with a history of headache and a blurred vision of two weeks’ duration. On clinical examination, he was plethoric and had a blood pressure of 190/100 mmHg. Ophthalmoscope examination (Figure 1) revealed normal bilateral disc with normal disc margin, a cup:disc ratio of 0:3, and congested retinal vessels with arteriovenous nicking and soft exudates along the arcades favoring a grade 2 hypertensive retinopathy. The rest of the clinical examination was unremarkable.

**Figure 2:**
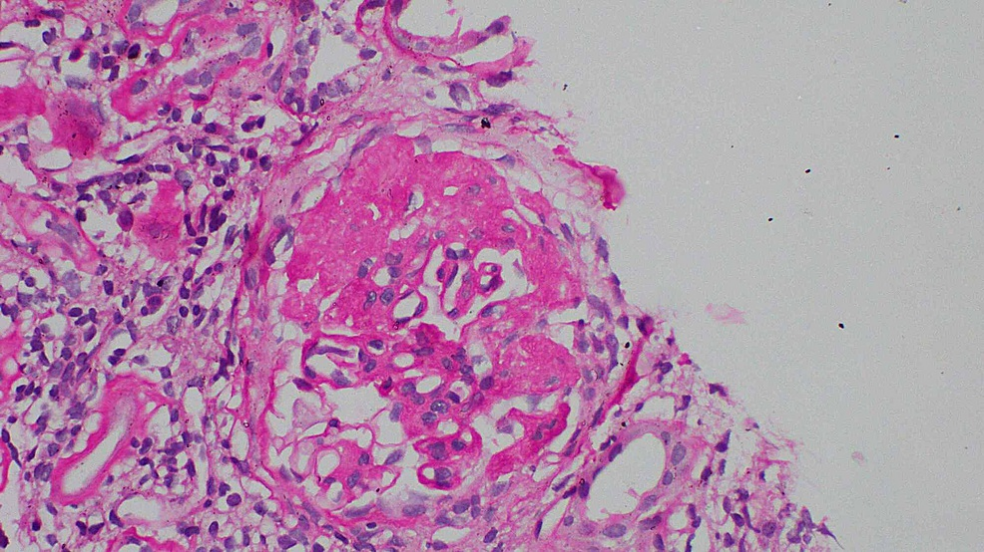
The glomerulus shows segmental sclerosis with viable capillaries in the rest (H&E: X400).

Initial investigation revealed a hemoglobin level of 20.4 g/dL with a hematocrit level of 56.9 and normal other cell lines (results of basic investigations are shown in Table 1). Urine full report showed 3+ proteinuria with 10-15 RBC/HPF, and further quantification revealed a urine protein:creatinine ratio of 9,190 mg/mg, which was well above the nephrotic range. His initial renal function was impaired with a serum creatinine of 3.19 mg/dL. Ultrasound imaging of the kidneys revealed increased echogenicity with normal-sized kidneys with loss of corticomedullary demarcation favoring chronic kidney disease. In view of nephrotic range proteinuria with active urinary sediment and normal-sized kidneys, the patient further underwent renal biopsy, which showed the features of IgA nephropathy with secondary FSGS (Figure 2). An extensive evaluation including ANA, Ds DNA, C3, C4, ASOT, hepatitis screening, and ANCA failed to identify a secondary cause of nephrotic range proteinuria.

**Table 1:** Results of basic investigations Abs., absolute; HCT, hematocrit; MCV, mean corpuscular volume; ESR, erythrocyte sedimentation rate; CRP, C-reactive protein; AST, aspartate aminotransferase; ALT, alanine aminotransferase

Biochemical investigations	On admission	At six weeks
Full blood count		
White cell count (4-10×10^9^/L)	7.36	9.32
Abs. neutrophils (2-7×10^9^/L)	6.57	6.2
Abs. lymphocytes (1-3×10^9^/L)	1.2	1.21
Abs. eosinophil (0.04-0.6×10^9^/L)	0.33	0.32
Abs. basophil (0.0-0.09 ×10^9^/L)	0.5	0.51
Hemoglobin (130-170 g/L)	20.4	14.5
HCT (0.40-0.50)	56.9	47
MCV (83-101 fL)	84	84
Red cell count (4.5-5.5×10^12^/L)	4.5	4.25
Platelets (150-410×10^9^/L))	258000	210000
Inflammatory markers		
ESR (first hour)	15	24
CRP (0-1.0 mg/L)	1	8
Electrolytes		
Sodium (135-145 mmol/L)	142	144
Potassium (3.5-5.0 mmol/L)	4.7	4.1
Calcium (2.1-2.5 mmol/L)	2.5	2.49
Phosphorus (1.2-1.4 mmol/L)	0.93	0.9
Ceratinine (mg/dL)	3.19	1.6
Blood urea (mmol/L)	9.9	4.2
Liver profile		
AST (0-45 U/L)	28	32
ALT (0-35 U/L)	36	34
Protein (6.4-8.3 g/dL)	6.7	7
Albumin (g/dL)	2.6	3.8
Globulin (g/dL)	3.1	3.2

He underwent a further evaluation to identify the cause for erythrocytosis in the background of euvolemia. Subsequent investigations showed normocytic normochromic crowded red cells with normal other cell lines and very low serum erythropoietin levels of <1.0 mIU/mL (normal range: 5.4-31 mIU/mL).

The rest of the investigations to find out a secondary cause of erythrocytosis failed to reveal any. As these findings did not reveal any causes of erythrocytosis, we have with an MNP test (including JACK2, Exon12mutation, CALR, MPL), which came out negative. Bone marrow examination showed erythroid hyperplasia with normal other cell lines. This extensive evaluation finally led to a diagnosis of IgA nephropathy with secondary FSGS.

The patient underwent two cycles of therapeutic phlebotomy. He was initiated with enalapril 10 mg twice a day and amlodipine 5 mg daily to control his blood pressure. In addition, he was prescribed aspirin and advised on adequate hydration. Following the nephrology evaluation, he was commenced with prednisolone 1 mg/kg (60 mg) daily and later on mycophenolate mofetil 500 mg twice a day was added.

At six weeks of treatment, serum creatinine had improved to 1.6 mg/dL from 3.19 mg/dL, and urine protein excretion was reduced to 1+. Hemoglobin remained in the normal range. Regular initial fortnightly and later monthly reviews were arranged by the medical, hematology, and nephrology team.

## Discussion

Erythrocytosis has an array of clinical presentations, such as hyperviscosity, thrombotic episodes, and hypertension [2]. Rarely it can be associated with renal manifestations. The renal diseases that are associated with erythrocytosis include IgA nephropathy, FSGS, MPGN, and RPGN. IgA nephropathy is the commonest and usually occurs in males [1].

There are many explanations for renal disease associated with erythrocytosis. The hyperviscosity in polycythemia leads to vascular microthrombi and glomerular capillary occlusion, thereby reducing GFR, which causes renal ischemia and leads to CKD [1]. These consequences can be prevented if the erythrocytosis is diagnosed early and treated accordingly. In addition, several cytokines and growth factors play an essential role in the progression of renal disease in erythrocytosis [1]. Of note, an abnormally upregulated mRNA expression of platelet-derived growth factor (PDGF) and insulin-like growth factor exacerbates the effect of polycythemia [1].

 In contrast to the above explanation, IgA-related syndrome is found to be associated with erythrocytosis [3]. IgA nephropathy is associated with an elevated level of polymeric IgA (plgA1) and circulatory IgA 1 complexes, which are involved in erythrocytosis [3].

The cause of erythrocytosis in our patient was not established. We excluded all the possible secondary causes. A very low serum erythropoietin level along with a negative MPN panel made a myeloproliferative disorder unlikely. Urinary loss of erythropoietin in nephrotic syndrome could explain the inappropriate low-level erythropoietin [4]. The possibility of erythropoietin receptor gene mutation was not excluded due to the unavailability of resources [5]. Finally, he was diagnosed to have IgA nephropathy associated with erythrocytosis and secondary FSGS.

The patient showed significant improvement in terms of renal function, control of hypertension, and stabilization of hemoglobin with the treatment. A prompt evaluation, early diagnosis, and appropriate interventions led to a successful treatment outcome.

## Conclusions

This case clearly demonstrates that IgA nephropathy could be associated with erythrocytosis. Although the association is unusual, prompt evaluation and appropriate interventions help improve renal function and control of blood pressure and also help stabilize the hemoglobin within the normal range.
